# Which factors influence the resort to surrogate consent in stroke trials, and what are the patient outcomes in this context?

**DOI:** 10.1186/s12910-015-0018-8

**Published:** 2015-04-24

**Authors:** Anne-Marie Mendyk, Julien Labreuche, Hilde Henon, Marie Girot, Charlotte Cordonnier, Alain Duhamel, Didier Leys, Régis Bordet

**Affiliations:** U1171-Department of Neurology, University of Lille, Inserm, Faculty of Medicine, Lille University Hospital, Lille, France; Clinical Research Federation, University of Lille, Lille University Hospital, Lille, France; EA2694-Department of Statistics, University of Lille, Faculty of Medicine, Lille University Hospital, Lille, France; Department of Emergency, Lille University Hospital, Lille, France; U1171-Department of Pharmacology, University of Lille, Inserm, Faculty of Medicine, Lille University Hospital, 1 place de Verdun, Lille cedex, F-59045 France

## Abstract

**Background:**

The provision of informed consent is a prerequisite for inclusion of a patient in a clinical research project. In some countries, the legislation on clinical research authorizes a third person to provide informed consent if the patient is unable to do so directly (i.e. surrogate consent). This is the case during acute stroke, when the symptoms may prevent the patient from providing informed consent and thus require a third party to be approached. Identification of factors associated with the medical team’s decision to resort to surrogate consent may (i) help the care team during the inclusion process and (ii) enable the patient’s family circle to be better informed (and thus feel less guilty) about providing surrogate consent.

**Methods:**

Patients included in the BIOSTROKE cohort (initially dedicated to the analysis of factors influencing stroke severity) were divided into two groups: those having provided informed consent directly and those for whom a third party (such as a family member) had provided surrogate consent. We compared the groups in terms of the initial clinical characteristics (age, gender, type of stroke, severity on the National Institutes of Health Stroke Scale (NIHSS), pre-stroke cognitive status according to the Informant Questionnaire on Cognitive Decline in the Elderly, and the stroke’s aetiology) and the functional and cognitive impairments (according to the NIHSS, the modified Rankin score (mRS) and the Mini Mental State Examination) on post-stroke days 8 and 90.

**Results:**

Three hundred and ninety five patients were included (mean ± SD age: 67 ± 15 years; 53% males). Surrogate consent had been obtained in 228 cases, and 167 patients had provided consent themselves. The patients included with surrogate consent were likely to be older and more aphasic, with a pre-existing cognitive disorder and more severe stroke (relative to the patients having provided consent). In terms of recovery, the patients included with surrogate consent had a worse functional prognosis (day 90 mRS ≥3: 57.6%, compared with 16.8% in patients having provided consent themselves; p < 0.0001) and a worse cognitive prognosis (day 90 MMS < 24: 15.4% and 4.8%, respectively; p < 0.002). The mortality rate was significantly higher in the surrogate consent group.

**Conclusions:**

We found that in addition to age, aphasia and stroke severity, pre-stroke cognitive status is a factor that should prompt the care team to consider requesting surrogate consent for participation in a clinical study. Given that the unfavourable outcome in patients with surrogate consent is often due to their initial clinical state (rather than inclusion in a trial *per se*), the issue of the family’s feelings of guilt (and how to avoid these feelings) should be further addressed.

## Background

In industrialized countries, stroke constitutes the leading cause of adult handicap, the second leading cause of dementia, and the third leading cause of death [1]. This impact on public health explains the ongoing development of basic research (using genetics, imaging, biomarkers, etc.) and clinical studies (on stroke prevention, acute-phase treatments, long-term outcomes, etc.). Research in this field is very active, as evidenced by the large number of studies registered on the *ClinicalTrials.gov website*. This type of study can only be performed if patients have provided their written, informed consent to participation. Even though the legislation in most countries includes some exceptions for obtaining consent to participation in clinical research on stroke (particularly as part of emergency care), informed consent remains the rule in most situations [2,3].

The provision of informed consent necessarily implies that the patient’s physical or cognitive state does not prevent him/her from making a reasoned, informed decision. However, patients with stroke symptoms do not always fulfil all the conditions for providing informed consent; this may prompt the investigator to approach a third party for “surrogate consent” (also referred to as an “exception from informed consent” [4,5]. Care teams will naturally be prompted to request surrogate consent when a patient presents with aphasia, altered consciousness or a sensorimotor impairment that prevents them from writing [4,6,7]. However, this procedure is less straightforward in other circumstances in which consent is not truly “informed”, such as moderate comprehension disorders, negligence, loss of visual field, and anosognosia. Furthermore, about 16% of stroke patients have pre-existing cognitive disorders; although this context might conceivably hinder the comprehension of study information, there are no literature data on the potential impact of these disorders [8].

Research on the provision of consent in stroke clinical trials has revealed that on average, only about a third of patients (between 15% and 47%, depending on the study) provide consent themselves [9-13]. For the other patients, surrogate consent is usually provided by a family member. Several studies have identified older patient age and greater initial stroke severity as being associated with the provision of surrogate consent. These studies have also emphasized the impact of certain symptoms (aphasia, impaired consciousness and anosognosia) and the type of stroke [9,11-13]. In contrast, neither the effect of pre-existing cognitive disorders nor the patient’s outcome has been assessed as a function of the type of consent. One study investigated the impact on families but did not examine the relationship with the patient’s functional prognosis [9].

The objectives of the present study were to (i) identify all the main factors influencing the care team’s decision to request surrogate consent and (ii) evaluate outcomes in patients with surrogate consent. We thus hoped to identify aspects that could alert the medical and paramedical staff in vascular neurology units and improve the provision of information to family members having provided surrogate consent to participation in a clinical study.

## Methods

### The study population

From among the BIOSTROKE cohort (initially dedicated to the analysis of factors influencing the severity of stroke), we prospectively recruited 395 patients with supratentorial stroke (ischemic stroke or spontaneous intracerebral haemorrhage) admitted to our university hospital’s stroke unit within 48 hours of symptom onset. Ischemic stroke was defined as clinical signs of focal cerebral dysfunction, lasting more than 24 hours, with no apparent cause other than vascular factors, and no sign of relevant primary intracerebral haemorrhage on brain imaging. Spontaneous intracerebral haemorrhage was defined as the sudden onset of an acute neurological impairment followed by confirmation of intraparenchymal haemorrhage on brain imaging. Patients with infratentorial lesions or haemorrhage caused by vascular malformations were excluded from the study. The study’s objectives and procedures were approved by the local independent ethics committee (reference: CP 05/15, Lille, France). Patients or close relatives provided written, informed consent.

### Data collected and definitions

Each patient’s demographic data, baseline characteristics, clinical information, examination data, final vascular diagnosis, medications, and follow-up information were collected using a structured questionnaire. All patients underwent an initial standardized evaluation, including their medical history, a physical examination, a routine blood biochemistry screen, and diagnostic testing. Fasted blood samples were collected on admission for lipid profile evaluation. Brain imaging (either MRI or a default CT scan) was performed immediately after sample collection. A 12-lead electrocardiogram (ECG), Doppler ultrasound examination of the neck, and transthoracic echocardiography were always performed. Other diagnostic procedures (such as magnetic resonance angiography, transcranial Doppler, 24-hour ECG monitoring, and digitized angiography) were performed in selected patients. Ischemic stroke subtypes were classified according to the Trial of Org 10172 in Acute Stroke Treatment criteria [14].

Arterial hypertension was defined as ongoing treatment with antihypertensive drugs; diabetes was defined as a fasting serum glucose level >7 mmol/L or ongoing use of antidiabetic drugs; dyslipidaemia was defined as fasting total cholesterol serum level >6.5 mmol/L, or the ongoing use of lipid-lowering drugs for a reason other than previous myocardial infarction; smokers were defined as patients smoking ≥10 cigarettes per/day at the time of inclusion or having ceased smoking within the previous five years; heavy drinkers were defined as patients with a weekly ethanol consumption of 300 g or more.

The stroke’s initial severity was measured according to the degree of neurological impairment on the National Institutes of Health Stroke Scale (NIHSS). The Glasgow Coma Score (GCS) and cognitive status (evaluated via a French translation of the Informant Questionnaire for Cognitive Decline in the Elderly; IQCODE) were also recorded on admission. Patients with a GCS score <15 were considered to have an initial alteration of the level of consciousness. Those with an IQCODE score >78 were considered to have cognitive impairment. Patients had a follow-up examination 8 and 90 days after admission, at which the NIHSS, the Mini Mental State Examination (MMSE) and the modified Rankin scale (mRS) were recorded. An NIHSS score >5 (severe clinical impairment), an MMSE score <24 (cognitive impairment) and an mRS score ≥3 (poor functional outcome) were considered as the worst possible stroke outcomes [15].

### Statistical analysis

Data are presented as the mean ± standard deviation (SD) or the median [interquartile range; IQR] for quantitative variables, and percentage (number) for categorical variables. Bivariate comparisons according to informed consent status were performed using Student’s *t* test for quantitative variables (Mann–Whitney *U* test was used for non-Gaussian distribution) and Chi-squared test (Fisher’s exact test was used when the expected cell frequency was <5) for categorical variables. The patient characteristics associated with informed consent status in bivariate analyses (p < 0.10) were introduced into a multivariate logistic regression analysis and subsequently used to adjust the between-group differences in stroke aetiology, severity and outcomes. We calculated the odds ratio (OR) [95% confidence interval (CI)] for each stroke outcome by using univariate and multivariate logistic regression analyses, with the patients having given informed consent as the reference group. Finally, we used a non-parametric analysis of covariance (adjusted for baseline NIHSS values) to compare the NIHSS change between baseline and 90 days after the stroke onset. Statistical testing was done at the two-tailed α level of 0.05. Data were analyzed using SAS software (version 9.3, SAS Institute Inc., Cary, NC, USA).

## Results

Of the 395 included patients (mean ± SD age: 67 ± 15 years, 53% males), 358 had suffered an ischemic stroke and 37 had suffered a haemorrhagic stroke. Informed consent to participation in the study was provided by the patient him/herself in 42% (n = 167) of the cases and by a surrogate in 58% (n = 228). The study population’s baseline characteristics are summarized in Table 1 as a function of informed consent status.

**Table 1:** **Characteristics of the study population and baseline stroke severity as a function of informed consent status**

	**Informed consent**		
	**Direct (n = 167)**	**Surrogate (n = 228)**	***P value***
Number of patients	N = 167	N = 228	
Age, y, mean ± SD	63.5 ± 14.8	70.2 ± 14.1	<0.0001
Men	99 (59.3)	112 (49.1)	0.046
BMI, kg/m^2^, mean ± SD	26.6 ± 5.1	26.6 ± 5.0	0.96
Qualifying event			
Ischemic stroke	156 (93.4)	202 (88.6)	0.10
Brain haemorrhage	11 (6.6)	26 (11.4)	
**Risk factors**			
Hypertension	102 (61.1)	144 (63.2)	0.67
Diabetes	29 (17.4)	45 (19.7)	0.58
Hypercholesterolemia	70 (41.9)	111 (48.7)	0.18
Smokers	59 (35.3)	56 (24.6)	0.020
Heavy drinkers	27 (16.2)	37 (16.4)	0.96
Physical activity	101 (61.2)	94 (41.4)	0.0001
History of stroke	13 (7.8)	36 (15.9)	0.016
History of ischemic heart disease	25 (15.0)	57 (25.0)	0.015
Heart failure	8 (4.8)	35 (15.4)	0.0009
Peripheral arteriopathy	8 (4.8)	14 (6.2)	0.54
**Stroke severity**			
GCS score <15	6 (3.6)	90 (39.5)	<0.0001
NIHSS score, median (IQR)	3 (1–5)	12 (6–19)	<0.0001
NIHSS score >5	41 (24.6)	178 (78.1)	<0.0001
Aphasia	31 (18.6)	119 (52.2)	<0.0001
Cognitive impairment (IQCODE > 78)	58 (37.9)	107 (49.8)	0.024

Values are quoted as the number (%), unless otherwise indicated.

Abbreviations. BMI: body mass index, GCS: Glasgow Coma Scale, IQCODE: Informant Questionnaire on Cognitive Decline in the Elderly, IQR: interquartile range, NIHSS: National Institutes of Health Stroke Scale, SD: standard deviation.

### The impact of patient- and stroke-related factors on the provision of *informed consent*

When compared with patients who provided informed consent themselves, patients with surrogate consent were older, less likely to perform regular physical activity, and more likely to be male, smoke and have a history of stroke, coronary artery disease or heart failure. The proportion of patients with surrogate consent increased gradually with each age quartile; using the lowest quartile as a reference, the ORs [95% CI] were 1.62 [0.92-2.86] for the second quartile, 2.29 [1.28-4.08] for the third quartile and 3.78 [2.08-6.86] for highest quartile. In a multivariate analysis, age (OR [95% CI] per 10-year increment: 1.25 [1.07-1.45]), no regular physical activity prior to the stroke (1.78 [1.15-2.73]) and a history of heart failure (2.71 [1.19-6.15]) were still associated with an elevated proportion of patients with surrogate consent.

**Figure 1:**
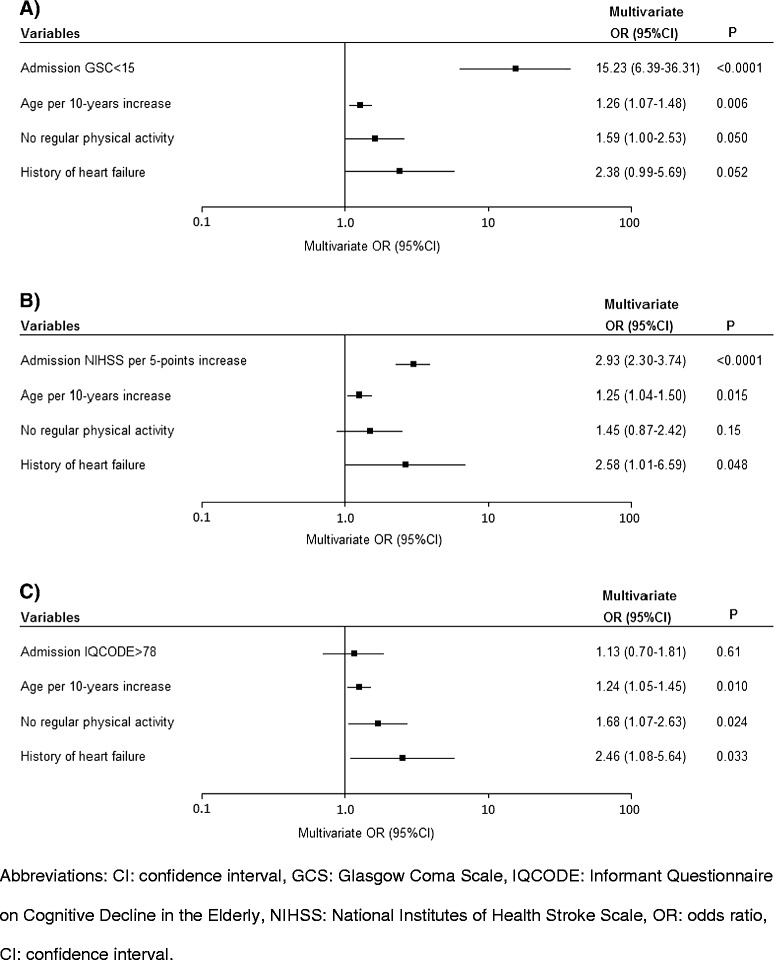
Multivariate analyses of the impact of stroke severity at admission (as assessed on the GCS **(A)**, the NIHSS **(B)** and the IQCODE **(C)**) on the likelihood of surrogate consent.

**Figure 2:**
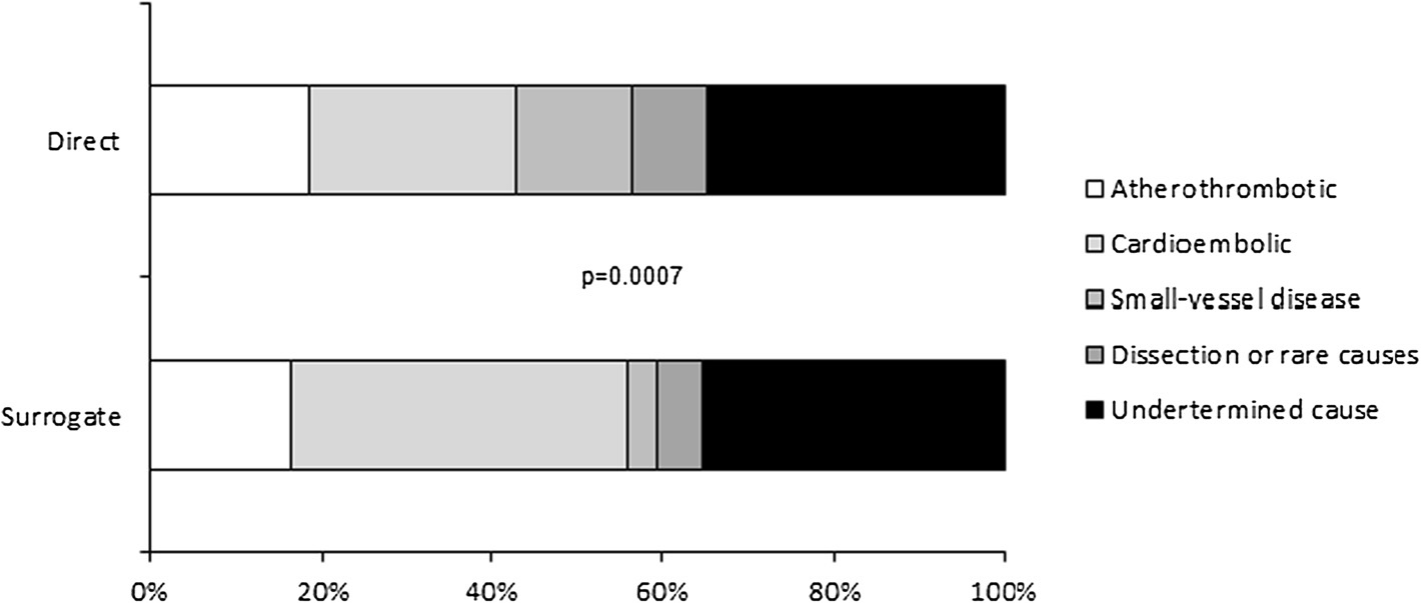
Ischemic stroke subtypes as a function of informed consent status.

In addition to demographic and risk factors and as expected, the stroke severity on admission (as assessed by the GCS, NIHSS or IQCODE scores) was strongly associated with informed consent status (Table 1). In a separate multivariate analysis (adjusted for age, physical activity and heart failure), an elevated proportion of patients with surrogate consent was associated with a higher stroke severity score (NIHSS > 5 or GCS < 15) (Figure 1). We specifically explored aphasia as a clinical symptom in patients with and without surrogate consent. Aphasia affected 19% (n = 31) of the patients having given informed consent and 52% (n = 119) of the patients with surrogate consent (*p* < 0.0001). As shown in Figure 2, there was a significant inter-group difference in ischemic stroke aetiology; surrogate consent was more frequent in patients with cardioembolic stroke and therefore less frequent in stroke due to small-vessel disease or rare causes. After adjustment for age, physical activity and heart failure, only the difference with respect to cardioembolic stroke was no longer statistically significant (*p* = 0.37). The difference related to stroke caused by small-vessel disease or rare causes was due to a significantly lower NIHSS score (median [IQR)]: 4 [1-6]) in these aetiologies than in other ischemic stroke aetiologies (median [IQR]: 7 [3-16]).

### Stroke prognosis as a function of *informed consent status*

**Figure 3:**
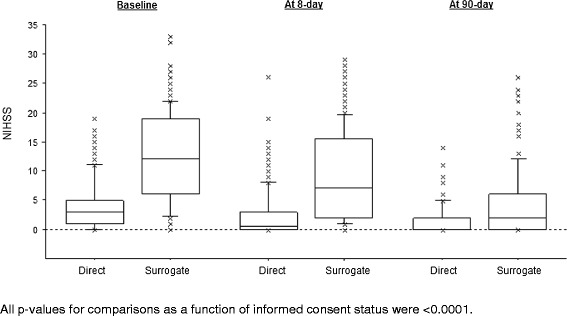
Distribution of the NIHSS scores at baseline and days 8 and 90 after stroke onset, as a function of informed consent status.

**Figure 4:**
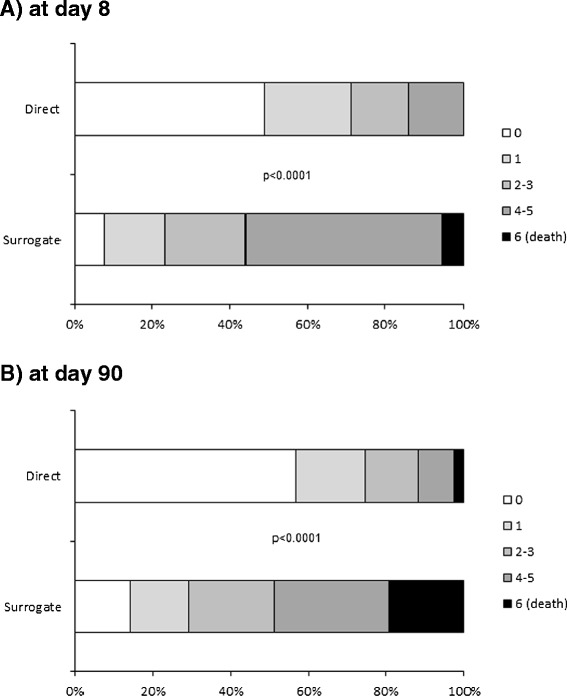
Modified Rankin scores at baseline and days 8 **(A)** and 90 **(B)** after stroke onset, as a function of informed consent status.

In agreement with the observed difference in stroke severity on admission, patients with surrogate consent had a worse stroke outcome on day 90 than patients who gave informed consent themselves (Table 2). As shown in Figure 3, patients with surrogate consent had a higher NIHSS score at both eight and 90 days, although the between-group difference at day 90 tended to decrease (relative to baseline). Of the patients still alive at day 90, the median [IQR] decrease in NIHSS from baseline was 5 [2-8] points in patients with surrogate consent and 2 [0–4] points in patients who gave informed consent (unadjusted and baseline NIHSS-adjusted p values: *p* < 0.0001 and *p* = 0.75, respectively). Only 29% (n = 62) of the patients with surrogate consent had an excellent outcome at day 90 (as defined by an mRS of 0 or 1), whereas the proportion was 75% (n = 120) for patients who gave informed consent themselves (Figure 4, *p* < 0.0001). The 90-day all-cause mortality rates were 19.3% (n = 41) for patients with surrogate consent and 2.5% (n = 4) for patients who gave informed consent themselves (*p* < 0.0001).

**Table 2:** **Poor stroke outcomes as a function of informed consent status**

	**Informed consent**					
	**Direct**	**Surrogate**	**OR (95% CI)**	***P***	OR (95% CI)*	P*
**Day 8 outcomes**	N = 166	N = 220				
NIHSS >5†	25 (15.0)	117 (51.3)	5.99 (3.64-9.85)	<0.0001	5.81 (3.46-9.76)	<0.0001
Cognitive impairment (MMSE < 24) ‡	21 (12.6)	58 (25.4)	2.37 (1.37-4.10)	0.002	1.95 (1.11-3.44)	0.021
Poor outcome (mRS ≥ 3)	36 (21.7)	144 (65.5)	6.84 (4.31-10.86)	<0.0001	5.76 (3.57-9.28)	<0.0001
All-cause death	0 (0.0)	12 (5.5)	-	-	-	-
**Day 90 outcomes**	N = 161	N = 212				
NIHSS >5†	10 (6.0)	44 (19.3)	3.75 (1.83-7.70)	0.0001	3.88 (1.85-8.15)	0.0003
Cognitive impairment (MMSE < 24) ‡	8 (4.8)	35 (15.4)	3.60 (1.63-7.99)	0.002	3.11 (1.38-7.01)	0.006
Poor outcome (mRS ≥ 3)	27 (16.8)	122 (57.6)	6.73 (4.10-11.04)	<0.0001	5.44 (3.24-9.12)	<0.0001
All-cause death	4 (2.5)	41 (19.3)	9.41 (3.30-26.87)	<0.0001	6.36 (2.18-18.58)	<0.0001

Values are quoted as the number (%), unless otherwise indicated. *adjusted for the patient’s age, physical activity before stroke and history of heart failure.

Abbreviations: MMSE: Mini Mental State Examination, mRS: modified Rankin score, NIHSS: National Institutes of Health Stroke Scale, OR: odds ratio, CI: confidence interval.

## Discussion

The proportion of stroke patients with surrogate consent to participation in our clinical study was well over 50%. Our present study results revealed that stroke patients who could not give consent were older, had a more severe stroke (often with aphasia) and were more likely to have a pre-existing cognitive impairment. The two groups differed in terms of their aetiological profile. Disease progression also differed, with a higher day 90 mortality rate and more severe functional handicap in the group of patients with surrogate consent.

Three studies have investigated the factors associated with surrogate consent. Two were based on a clinical evaluation [9,11] and the third was based on an imaging assessment [12]. All three were drug trials: two compared tissue plasminogen activator with a placebo and the third evaluated MRI imaging and/or a neuroprotective drug. In contrast, the present study was a non-interventional, physiopathological investigation; nevertheless, the proportion of patients with surrogate consent was similar to that observed in drug trials. This observation suggests that the type of trial does not influence the investigators’ judgement of a patient’s capacity to provide consent him/herself. One limitation of the present study relates to the fact that we did not obtain any data on (i) the criteria associated with a physician’s decision to resort to surrogate consent or (ii) the characteristics of patients and surrogate who refuse to give consent. Our results are also in good agreement with a previous report showing that the probability of obtaining surrogate consent was higher for low-risk and moderate-risk studies than for high-risk studies [10]. One could perhaps consider that the non-interventional nature of the present study was a limitation, since it did not reflect the situation in interventional studies (i.e. in which provision of consent is crucial for ethical and regulatory reasons). However, it must be noted that according to the literature, 50% of subjects do not understand the study’s objectives sufficiently well - although this does not influence the decision to participate or not [10].

Our study identified a number of factors that had already been associated with surrogate consent in the literature: age, the initial severity of the stroke, the presence of aphasia and an altered level of consciousness [9,11-13]. A secondary objective of our present work was to raise the issue of the potential impact of the patient’s pre-existing cognitive state on his/her ability to give consent. Previous research has established that 16% of patients have altered cognitive function prior to stroke, which increases stroke severity and aggravates cognitive disorders [8]. In the present study, an abnormal score for the Informant Questionnaire on Cognitive Decline in the Elderly was associated with lower ability to provide consent. The existence of pre-existing cognitive disorders will necessarily alter the patient’s ability to make judgements, to understand and remember information and thus to provide truly informed consent. Pre-existing cognitive disorders are less obvious than other factors associated with surrogate consent and should be screened for systematically prior to a request for informed consent.

In addition to these statistical data (which define an average profile), it is also noteworthy that consent was obtained from six patients with an abnormal Glasgow score. Likewise, consent was obtained from 31 patients with aphasia and 58 patients with pre-existing cognitive disorders, despite the fact that the study was being performed by a care team with significant experience of clinical trials. One can question the validity of this consent, in view of the comprehension difficulties that form part of phasic and cognitive disorders - even though aphasia was less severe in aphasic patients having given their consent directly (data not shown) [6,7]. In contrast, one can consider that it would be unfair not to include these patients in trials because (i) they constitute a group with clear treatment needs (in view of the poor prognosis) and (ii) excluding them from the decision-making process will reduce their personal autonomy. Our data show that it was possible to include patients with aphasia, and thus supports Stein et al.’s suggestion that the use of surrogate consent should be avoided because it compromises the patient’s personal independence [16].

The present study’s third objective was to compare the prognoses in patients who had been able to provide consent and those with surrogate consent. Worsening over time was clearly more severe in patients with surrogate consent. Of course, there is no direct, causal relationship between the change over time and the ability to provide consent; the stroke’s initial severity is the link between these two factors. We did not interview the people who gave surrogate consent on how they felt about taking this decision; this constitutes a study limitation. Nevertheless, in two studies of the family’s feelings, family members considered that they had made the decision to give surrogate consent freely and had not been concerned about the possible impact of refusal on the patient’s subsequent care. Despite the perceived burden of responsibility, the great majority of the families stated that the decision was easy to take [11,17]. However, one cannot rule out the possibility that some families do establish a causal relationship between their provision of surrogate consent to the patient’s inclusion in a trial and a subsequent poor outcome (persistence of the deficiency, dependence, etc.), even though this has no medical or scientific justification. The results of a recent study emphasized that rates of agreement to possible exemption from informed consent were lower than expected in patients who had already participated in clinical research [18]. Now that social networks and discussion forums deal with medical questions extensively, it is important to ensure that the level of approval for surrogate consent to inclusion in acute stroke trials remains as high as it currently is [19]. Even though there are legal measures for exception from informed consent, it is possible that families will attribute the patient’s poor prognosis to participation in the trial and thus give surrogate consent less readily. This would exclude the most severe cases of stroke from clinical trials and thus limit the latter’s value [20,21]. We know that this point remains questionable because our study was purely descriptive and did not collect qualitative data on the family’s feelings. Nevertheless, the fact that the analysis used in the present study was purely observational (i.e. with the absence of any medical acts likely to have an impact on the stroke’s prognosis) shows that the patient’s prognosis is related to the initial severity of the stroke, rather than the type of consent. Indeed, the initial severity of the stroke influences both the ability to provide consent and the functional prognosis. Since the unfavourable outcome in patients who are unable to give direct consent is often due to their initial state (and not their inclusion in a trial *per se*), the question of the family’s feeling of guilt (and how to avoided that guilt) should be further addressed. There is a need for more relevant information on the impact of the decision to provide surrogate consent to a family member’s inclusion in a study.

## Conclusions

Provision of surrogate consent by a third party remains essential in stroke patients. It is clearly necessary for the inclusion of older patients with pre-existing cognitive disorders, a severe stroke and/or aphasia. Study investigators must remain vigilant because consent may sometimes be obtained directly from this type of patient - even though the issues of personal autonomy and the patient’s role in the decision to participate in a clinical study must be considered. Families should be informed that the risk of a poor prognosis is unrelated to their approval of inclusion, since the worse prognosis in this type of patient is related to the disease and not to the type of consent. Provision of this information should help the family to avoid feelings of guilt.
